# Titania-Coated Alumina Foam Photocatalyst for Memantine Degradation Derived by Replica Method and Sol-Gel Reaction

**DOI:** 10.3390/ma13010227

**Published:** 2020-01-04

**Authors:** Zrinka Švagelj, Vilko Mandić, Lidija Ćurković, Martina Biošić, Irena Žmak, Mattia Gaborardi

**Affiliations:** 1 Department of Materials, Faculty of Mechanical Engineering and Naval Architecture, University of Zagreb, Ivana Lučića 5, 10000 Zagreb, Croatia; zrinka.svagelj@fsb.hr (Z.Š.); irena.zmak@fsb.hr (I.Ž.); 2 Department of Inorganic Chemical Technology and Non-Metals, Faculty of Chemical Engineering and Technology, University of Zagreb, Marulićev trg 20, 10000 Zagreb, Croatia; 3 Department of Analytical Chemistry, Faculty of Chemical Engineering and Technology, University of Zagreb, Marulićev trg 20, 10000 Zagreb, Croatia; mperisa@fkit.hr; 4 Elettra Sincrotrone Trieste S.C.p.A., Strada Statale 14, 4149 Basovizza, Trieste, Italy; mattia.gaboardi@elettra.eu

**Keywords:** Al_2_O_3_ foam, TiO_2_, photocatalysis, memantine, LED lamp

## Abstract

In the present work, alumina (Al_2_O_3_) foam was prepared by the replica method where a polyurethane (PU) foam (30 pores per inch (ppi)) template was impregnated with a 60 wt.% Al_2_O_3_ suspension. Sintered Al_2_O_3_ foam was used as substrate for the deposition of sol-gel derived titania (TiO_2_) film using dip coating. For the preparation of TiO_2_ sol, titanium(IV) isopropoxide (Ti-iPrOH) was used as the precursor. The common problem of qualification and quantification of a crystalline coating on a highly porous 3D substrate with an uneven surface was addressed using a combination of different structural characterization methods. Using Powder X-ray Diffraction (PXRD) and synchrotron Grazing Incidence X-ray Diffraction (GIXRD) on bulk and powdered Al_2_O_3_ foam and TiO_2_-coated Al_2_O_3_ foam samples, it was determined Al_2_O_3_ foam crystallizes to corundum and coating to anatase, which was also confirmed by Fourier Transformed Infrared Spectroscopy (FTIR). Scanning Electron Microscopy with Energy Dispersive X-ray Spectroscopy (SEM/EDS) revealed the structural and microstructural properties of the substrate and coating. Differential Thermal Analysis (DTA) and Thermogravimetric Analysis (TGA) were used to clarify the evolution of the porous microstructure. The Al_2_O_3_-TiO_2_ composite was evaluated as a photocatalyst candidate for the degradation of the micropollutant medication memantine. The degradation rate was monitored using a light-emitting diode (LED) lamp operating at electromagnetic (EM) wavelength of 365 nm. The photocatalytic activity of sol-gel-derived TiO_2_ film immobilized on the Al_2_O_3_ foam was compared with commercially available TiO_2_ nanoparticles, P25-Degussa, in the form of a suspension. The levels of memantine were monitored by High-Performance Liquid Chromatography–Tandem Mass Spectrometry (HPLC–MS/MS). The efficiency and rate of the memantine photodegradation by suspended TiO_2_ nanoparticles is higher than the TiO_2_-coated Al_2_O_3_ foam. But, from the practical point of view, TiO_2_-coated Al_2_O_3_ foam is more appropriate as a valuable photocatalytic composite material.

## 1. Introduction

Engineered foams can be manufactured from ceramics, glasses, metals and polymers. The first ceramic foam was developed and patented by Schwartzwalder and Somers in 1963 [1]. Since then, the number of publications and patents in the field of ceramic foams shows exponential growth. Ceramic foams have numerous pores and high specific surface, boosting their range of applicability, such as in filters for metal melts, exhaust gases, hot corrosive industrial gases and other solid–fluid separation processes, high-temperature thermal insulation and heat exchangers, bone replacement tissue engineering, lightweight and other structural products, catalyst supports and catalysts, etc.

Ceramic foams have been prepared using several methods such as direct foaming [2,3,4,5,6,7], sacrificial template or fugitives [8,9,10,11,12], replica methods [1,13,14,15,16] and partial sintering [17,18]. Ceramic foam derived using the abovementioned methods is chemo-thermo-mechanically suitable for further processing. In the present research, the photocatalyst material was immobilized on the available substrate surface. For immobilization of a photocatalyst different methods have been considered. Advanced deposition methods include Chemical Vapor Deposition (CVD), Plasma Enhanced Chemical Vapor Deposition (PECVD), physical deposition methods such as Electron Beam and Thermal Evaporation Deposition, Plasma Enhanced Chemical Vapor Deposition (PECVD), Pulsed Laser Deposition (PLD), Cold Plasma Discharge (CPD), Radio Frequency Magnetron Sputtering (RFMS) and Electrophoretic Deposition (EPD). Chemical deposition methods include spray coating, dip coating, spray pyrolysis, hydrothermal and sol-gel processing [19,20,21,22,23,24,25]. Among the aforementioned methods, chemical deposition methods stand out as more suitable due to lower economic and practical demands for the deposition onto unevenly shaped substrates, such as ceramic foam. Among these, the sol-gel method displays additional advantages including the ability to easily control the preparation of material with good homogeneity and stoichiometry using affordable equipment. Additionally, the sol yield is appropriate for coating of substrates with various sizes and shapes at comparatively low processing temperatures. The preparation conditions heavily affect the structure and morphology of the deposited films, which is also true for TiO_2_ films. Finally, sol-gel derived composites, including TiO_2_ films, normally show excellent photocatalytic properties [5].

Over the past decades, considerable attention has been paid to the photodegradation of organic pollutants from air, water and wastewaters using the oxide-based semiconducting photocatalysts. TiO_2_ emerged as a benchmark material due to its high photocatalytic activity, excellent physical and chemical stability, nontoxicity, abundance and adequate price [19,20,21]. Photocatalysis is one of the important Advanced Oxidation Processes (AOP’s) where highly reactive hydroxyl radicals (OH) react with a large variety of environmental contaminants, and degrade them into species like CO_2_, H_2_O, or mineralize them into harmless inorganic anions. TiO_2_ is a wide bandgap semiconducting oxide that occurs in three crystalline polymorphs: anatase (tetragonal), rutile (tetragonal) and brookite (orthorhombic). Among these, anatase is characterized by the indirect band gap with the energy of 3.23 eV and the absorption edge at 386 nm in the near ultraviolet (UV) range, all contributing to high photoactive applicability [26,27]. Rutile possesses a narrower band gap at 3.02 eV with an absorption edge at 416 nm in the visible (VIS) range. Both forms are interesting for specific photocatalytic applications while brookite is less common as it is a high-temperature polymorph. Generally, two types of photocatalytic reactors can be found in literature: slurry photoreactors (suspended photocatalyst particles) and fixed-bed photoreactors (immobilized photocatalyst particles or films on a surface of adequate planar substrates such as glass, quartz, stainless steel, polymers, or porous substrates such as pumice stone, clay, ceramics, polymeric materials, zeolites, carbon nanotubes, graphene oxide, fibers, etc.) [28,29,30,31]. In practice, immobilized catalyst photoreactors are preferred for advanced wastewater treatment as they avoid the complex separation step which is needed in the case of slurry photoreactors. Immobilized catalyst photoreactors also enhance the catalyst lifetime. Yet immobilized catalyst photoreactors have disadvantageously low interfacial surface areas and therefore low activity, and are difficult to scale-up [22,23]. Hence, the advancements in the immobilized catalyst photoreactors area focus into increasing specific surface and/or the reactivity and/or pollutant affinity of the photocatalysts, while retaining strong adherence between the catalyst and the substrate.

This paper reports about new upgrades in following areas. (i) Generation of a highly porous Al_2_O_3_ ceramic foam by a simple and low-cost replica method based on the impregnation of the polyurethane (PU) sponge with a highly concentrated Al_2_O_3_ suspension. (ii) Deposition of a photocatalytic TiO_2_ film on the Al_2_O_3_ foam substrate by means of sol-gel dip coating. (iii) Addressing of a common problem of qualification and quantification of a crystalline coating on a highly porous 3D substrate with uneven surface. (iv) Characterization and investigation of the photocatalytic activity of the prepared TiO_2_-coated Al_2_O_3_ foam on a model pollutant such as memantine, a drug used in Parkinson’s disease and movement disorders treatment and recently demonstrated to be useful also for treatment of dementia syndrome.

## 2. Materials and Methods

### 2.1. Chemicals and Reagents

Commercial Al_2_O_3_ powder CT 3000 SG (Almatis, Ludwigshafen, Germany) with 99.78% purity and mean particle size of 0.5 µm was used for the preparation of highly concentrated aqueous ceramic suspension. In order to stabilize the ceramic suspension and to achieve good coating behavior, commercial organic additives were used: carbonic acid-based polyelectrolyte Dolapix CE 64 (Zschimmer & Schwarz, Chemie, Lahnstein, Germany), polyvinyl alcohol (PVA, 99+ % hydrolyzed, Sigma–Aldrich, SAD, St. Louis, MO, USA) and Foamaster MO 2111 (BASF, Ludwigshafen, Germany). Polyurethane foam (Rekord-tim, Oriovac, Croatia) with the pore density of 30 pores per inch (ppi) was used as a template for the preparation of Al_2_O_3_ foam photocatalyst support.

For the preparation of TiO_2_ sol (colloidal solution) the following analytical grade reagents were used: titanium(IV) isopropoxide (Ti-iPrOH, Ti(OCH(CH_3_)_2_)_4_, 98%, Sigma–Aldrich, St. Louis, MO, USA), *i*-propyl alcohol (iPrOH, C_3_H_7_OH, 99.9%, Gram-Mol, Zagreb, Croatia), acetylacetone (AcAc, CH_3_(CO)CH_2_COCH_3_, 99%+, VWR International, Radnor, PA, USA) and nitric acid (NA, HNO_3_, 65%, Carlo Erba Reagents, Val-de-Reuil, France).

For photocatalysis the following analytical grade reagents were used: analytical standards of memantine hydrochloride (3,5-Dimethyl-1-adamantanamine hydrochloride, C_12_H_21_N HCl, 98%+, CAS: 41100-52-1, Sigma–Aldrich, USA), acetonitrile (C_2_H_3_N, ≥ 99.9%, Kemika, Zagreb, Croatia), formic acid (CH_2_O_2_, ≥ 98%, Kemika, Zagreb, Croatia) and ultrapure water, Millipore Simplicity UV system (Millipore Corporation, Burlington, MA, USA).

Commercially available titania nanoparticles catalyst from P25-Degussa, with purity 99.9%, was used as received for the comparison of photocatalytic activity of prepared TiO_2_ film on Al_2_O_3_ foam substrate. The P25-Degussa was used without further modification, i.e., it is mostly in the anatase form (75–80% anatase and 20–25% rutile), nonporous, with a reactive surface (BET) area of 50 to 54 m^2^ g^−1^, corresponding to a mean particle size of around 30 nm [32].

### 2.2. Preparation and Characterization of Al_2_O_3_ Suspension

Aqueous Al_2_O_3_ suspension with 60 wt.% of solid loading was prepared by dispersing ceramic powder and dissolving organic additives in distilled water. Firstly, the following amounts of organic additives (based on the amount of Al_2_O_3_ powder) were dissolved in distilled water while stirring: 0.4 wt.% of Dolapix CE 64 was used as dispersant that provides a production of ceramic suspension with a high solids content, 3.5 wt.% of PVA was used as temporary binder and plastifying agent to improve green and dry breaking strength and to increase a suspension viscosity and 0.5 wt.% of Foamaster MO 2111 (BASF, Ludwigshafen, Germany) was used as defoaming agent to prevent foaming during the replica process.

Afterwards, Al_2_O_3_ powder was added into the prepared solution and the mixture was homogenized in the planetary ball mill grinding jar (PM 100, Retsch, Haan, Germany) with ten alumina balls (10 mm diameter) for 60 min at a rotation speed of 300 rpm. After the homogenization, the Al_2_O_3_ balls were separated from the suspension and the air bubbles were removed from the suspension by ultrasonic treatment for 10 min in an ultrasonic bath (Bransonic 220, Branson Ultrasonics, Danbury, CT, USA).

The prepared Al_2_O_3_ suspension showed an apparent viscosity of 672 mPa·s at 100 rpm, measured by the rotational viscometer DV-III Ultra (Brookfield Engineering Laboratories, Middleborough, MA, USA) using a small sample chamber and spindle SC4-34. The viscosity measurement was conducted at a constant temperature of 25 ± 1 °C.

### 2.3. Preparation of Al_2_O_3_ Foam

Al_2_O_3_ foam was prepared by the above-mentioned replica method employing the 30 ppi polyurethane (PU) foam as a template. The PU foam was cut in the form of a ring with outer diameter of 90 mm, inner diameter of 40 mm and thickness of 15 mm (for the photocatalytic experiment) and in form of a cube (for the structural and mechanical characterization methods) using a hot wire cutter Thermocut 230/E (Proxxon Micromot, Luxemburg). PU foam ring and cubes were immersed into previously described 60 wt.% Al_2_O_3_ suspension and compressed three times to allow suspension to completely impregnate the PU foam surface. The excess suspension was removed using a centrifuge to prevent the blocking of the pores and to obtain a uniform coating. Acceptable coverage of the template was reached after four impregnation–centrifuging cycles, with air drying at 80 °C for 30 min between each impregnation step.

**Figure 1:**
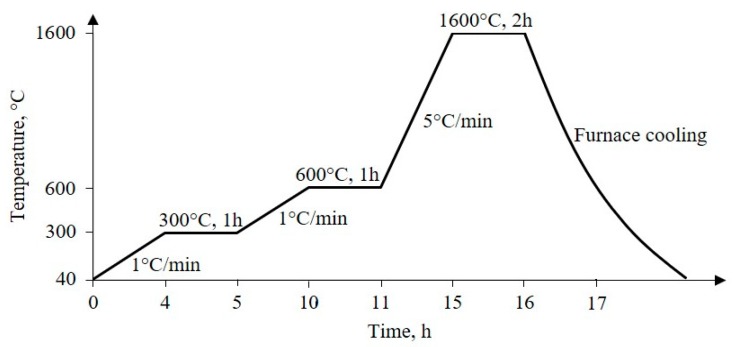
Scheme of sintering regime of Al_2_O_3_ foam.

In order to adjust the sintering process of Al_2_O_3_ foams, the course of the thermal decomposition of the 30 ppi PU foam was investigated using Differential Thermal Analysis (DTA) and Thermo-Gravimetric Analysis (TGA). For this purpose, the simultaneous DTA/TGA device STA409 (Netzsch, Selb, Germany) was used. A platinum crucible was filled with approximately 5 mg of material and heated up to 900 °C at a heating rate of 10 °C/min in synthetic air flow of 30 cm^3^/min, with corundum powder used as a reference. According to the PU foam thermal decomposition results, the heat treatment process was defined, as shown in Figure 1.

Firstly, a slow heating rate of 1 °C/min to 600 °C with 1 h holding period at 300 °C and at 600 °C was applied to prevent the structure from collapsing during the burnout of the PU foam and other organic matter. Subsequently, a heating rate of 5 °C/min was applied to the final sintering temperature of 1600 °C. After the furnace cooling, the consolidated Al_2_O_3_ foam, which is the replica of the PU foam template, was obtained and used as the catalyst substrate.

### 2.4. Deposition of Nanostructured TiO_2_ Film on Al_2_O_3_ Foam Substrate

The nanostructured TiO_2_ film was deposited on the Al_2_O_3_ foam ring substrate by sol-gel assisted dip coating. For that purpose, TiO_2_ sol was prepared by mixing titanium(IV) isopropoxide as a precursor, *i*-propyl alcohol as a solvent, acetylacetone as a chelating agent and nitric acid as a catalyst. The molar ratio of these reactants was Ti-iPrOH:iPrOH:AcAc:NA = 1:35:0.63:0.015 [24]. After two days of aging under ambient conditions, the prepared TiO_2_ sol was used in the coating process by dipping the Al_2_O_3_ foam substrates into the TiO_2_ sol for 10 min followed by drying at 80 °C for 60 min. The coating process was performed two times. Finally, the TiO_2_-coated Al_2_O_3_ foam substrates were calcined at 450 °C for 1 h with a heating rate of 3 °C/min.

### 2.5. Characterization of Al_2_O_3_ Substrate and TiO_2_-Coated Al_2_O_3_ Foam

The structure of the TiO_2_-coated Al_2_O_3_ foam was investigated by diffraction techniques. XRD fixed step scans were performed using the XRD6000 (Shimadzu, Japan) using 0.02° 2θ steps in the range 10–70° 2θ with holding time of 0.6 s at accelerating voltage of 40 kV and current of 30 mA. Samples were recorded as received (bulk cube), and ground into powder. In addition, GIXRD spectra were recorded using 8 keV synchrotron radiation on the MCX beamline of the Elettra Synchrotron facility in Trieste (Elettra, Basovizza, Italy) [33]. As received specimens were fixed on the sample-stage plate in the 4-axis Huber goniometer and the position was adjusted using z-scan and θ-scan. The fixed step measurements were performed using 0.01° 2θ-steps in the range 20–30° 2θ with counting time of 0.2 s using spot (300 × 1000 μm) analysis.

Infrared spectroscopy measurements were performed using Vertex 70 FTIR device (Bruker, Billerica, MA, USA) in the attenuated total reflectance (ATR) mode in the spectral region 4000–400 cm^−1^ with resolution of 1 cm^−1^ as an average of 32 scan.

Scanning Electron Microscopy (SEM) and Energy Dispersive Spectroscopy (EDS) were used to investigate the microstructural and compositional aspects of the Al_2_O_3_ foam substrate and TiO_2_-coated Al_2_O_3_ foam. For that purpose, a Vega 3SE EasyProbe electron microscope device (Tescan, Brno, Czech Republic) additionally equipped with an energy dispersive X-ray spectrometer INCA X-sight (Oxford Instruments, Abington, UK) was used. Before scanning, the samples were fixed on an appropriate holder using carbon tape adhesive and silver paste while the conductivity of the samples was ensured by sputtering gold particles using SC 7620 (Quorum, Lewes, UK) sputter coater.

Images obtained by optical microscopy (Olympus BX51, Tokyo, Japan) were analyzed using software Motic Images Plus 3.0 to determine the average pore size (face diameter) and the average strut thickness of Al_2_O_3_ foam substrate and TiO_2_-coated Al_2_O_3_ foam.

The compressive strength (*σ*) of Al_2_O_3_ foam substrate and TiO_2_-coated Al_2_O_3_ foam was measured using universal testing machine (WPM VEB Thüringer Industriewerk, Rauenstein, Germany) with a cross head speed of 5 mm/min. The compressive strength of ten samples with dimensions of approximately 15 × 15 × 15 mm was determined from the maximum load at failure and the loaded surface area.

### 2.6. Photocatalytic Experiment

The TiO_2_-coated Al_2_O_3_ foam ring was immersed into 100 mL of aqueous solution of memantine with a concentration of 10 mg/L. Memantine solution was prepared by dissolving the memantine hydrochloride powder in deionized water of MilliQ quality.

**Figure 2:**
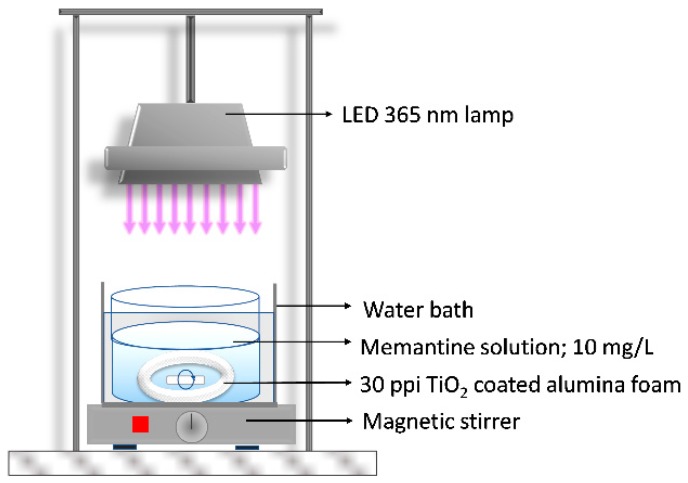
Scheme of photoreactor system.

The TiO_2_-coated Al_2_O_3_ foam was placed at the bottom of the reactor (Figure 2) and after that 100 mL of memantine solution of concentration 10 mg/L was added. During the experiment the memantine aqueous solution was constantly mixed using a magnetic stirrer and the distance between the light-emitting diode (LED) lamp with a radiation peak at 365 nm and the memantine aqueous solution was 20 cm. The photocatalytic activity of sol-gel derived TiO_2_ film immobilized on the Al_2_O_3_ foam was compared with commercially available TiO_2_ nanoparticles P25-Degussa in the form of a suspension. Therefore, 100 mL of memantine solution of concentration 10 mg/L and 100 mg of TiO_2_ nanoparticles P25-Degussa were added in the reactor (Figure 2). After that, the same UV-A LED lamp irradiation test was performed. The suspended TiO_2_ nanoparticles catalyst used for the experiment had to be removed by filtration in all collected samples for the analysis of memantine concentration. Prior to irradiation (for both photocatalytic experiments: one in the form of a film on alumina foam and the other with suspended TiO_2_ nanoparticles), the systems were magnetically stirred in the dark (“dark adsorption” experiment) for 60 min to ensure the equilibrium of memantine adsorption–desorption on the surface of TiO_2_. Also, the photolytic degradation test of memantine under UV-A radiation (365 nm) (without TiO_2_ in form film or suspension) was performed. All experiments of memantine degradation were carried out at a temperature of 23 °C ± 0.2 °C using a water bath with thermostat control. Samples for the analysis were collected in defined time intervals and stored in dark under −4 °C until analysis. All of the experiments were triplicated with errors below 4%.

The degradation rate of memantine was analyzed by High-Performance Liquid Chromatography–Tandem Mass Spectrometry (HPLC–MS/MS), which was performed with an Agilent Series 1200 HPLC system (Santa Clara, USA) coupled with an Agilent 6410 triple-quadrupole mass spectrometer equipped with an ESI interface. Chromatographic separation was undertaken on a Kinetex C18 column (100 mm × 2.1 mm, particle size 2.5 μm) supplied by Phenomenex, USA. The mobile phase comprised MilliQ water with 0.1% formic acid as eluent A and acetonitrile with 0.1% formic acid as eluent B. The composition of 50% organic phase was constant at a flow rate of 0.2 mL/min throughout the analysis. An injection volume of 5 μL was used in all analyses. The analysis was done in positive ion mode under the following conditions: drying gas temperature 350 °C; capillary voltage 4.0 kV; drying gas flow 11 L/min and nebulizer pressure 35 psi. Instrument control, data acquisition and evaluation were done with Agilent MassHunter 2003–2007 Data Acquisition for Triple Quad B.01.04 (B84) software (Agilent, Santa Clara, CA, USA).

## 3. Results and Discussion

### 3.1. Thermal Evolution of PU Foam

**Figure 3:**
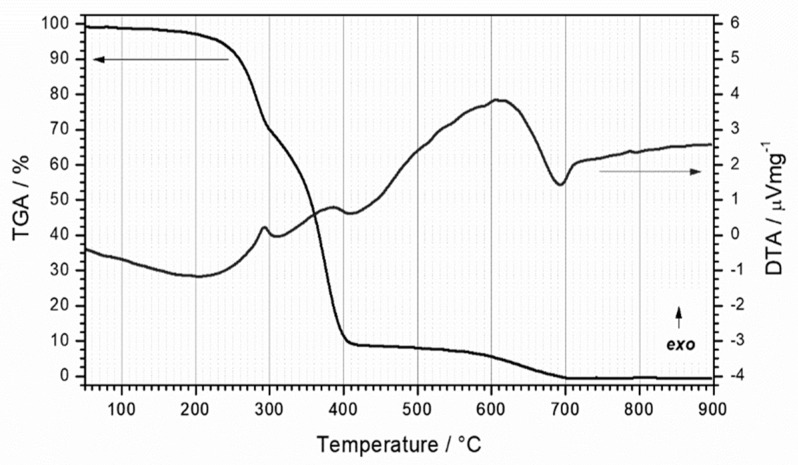
Differential Thermal Analysis (DTA) and Thermo-Gravimetric Analysis (TGA) curves of the 30 pores per inch (ppi) PU foam.

The course of the thermal decomposition of the PU foam started with a faint release of the adsorbed moisture and organic matter. This feature was characterized by a minute mass loss in the thermal region up to 150 °C and with no thermal effect (Figure 3). The non-flaming thermal degradation of some PU products may begin in the thermal region as low as 150–180 °C [34]. However, the flash ignition point is commonly above 300 °C and below 400 °C. In the thermal region above 250 °C decomposition onset may be observed. This temperature corresponds to 5% mass loss, which is usually considered to be the initial temperature of the sample decomposition process [35]. The decomposition of PU occurs in two well defined stages [36,37]. It is characterized with significant mass loss over the temperature region 250–400 °C and accompanied with clearly observable exothermic effects. The burning of the PU is basically an oxidation of the polymer into carbon monoxide, carbon dioxide, hydrogen cyanide and nitrogen dioxide. At 350 °C, at 50% mass loss, the decomposition of soft segments was observed. Thereafter, the mass loss rate decreased, but it was still present in the temperature range of 400–700 °C. Specifically, despite the polymer being degraded, the burning of the carbonaceous product still took place. This effect was weak in intensity and dispersed over the temperature range, so it was accompanied only with a weak exothermic thermal effect. The temperature region above 700 °C to 900 °C shows only a minute mass loss and no thermal effects and can be considered as a region without mass loss. The sample was fully decomposed and only char traces remained (Figure 3). The PU material was confirmed to be suitable for the use as a template for the replica method.

### 3.2. Properties of Al_2_O_3_ Foam Substrate and TiO_2_-coated Al_2_O_3_ Foam

Total porosity (*ϕ*) of sintered Al_2_O_3_ foam substrate was calculated using Equation (1):

(1) $$\phi =1-\frac{\rho }{\rho^{*}}$$

where *ρ* is the bulk density of Al_2_O_3_ foam substrate and TiO_2_-coated Al_2_O_3_ foam calculated from the mass to bulk volume ratio and *ρ* * is the theoretical density of Al_2_O_3_ (3.986 g/cm^3^). Total porosity mean values were calculated from ten porosity measurements of Al_2_O_3_ foams. The results of density, porosity, average strut thickness, average pore size (face diameter) and compressive strength of are listed in Table 1. The obtained results of the compressive strength are consistent with previous findings [38,39]. As can be seen from data shown in Table 1, there were no significant differences in the morphological properties and compressive strength between Al_2_O_3_ foam substrate and TiO_2_-coated Al_2_O_3_ foam.

### 3.3. Morphology of Al_2_O_3_ Foam Substrate and TiO_2_-coated Al_2_O_3_ Foam

**Figure 4:**
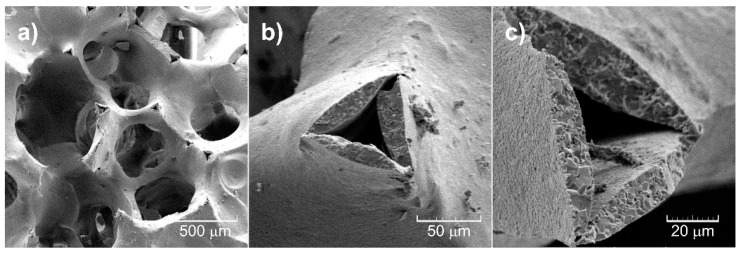
SEM images of the 30 ppi Al_2_O_3_ foam substrate: (**a**) network, (**b**) bridges, (**c**) inner porosity visible on micrographs at high magnification.

The morphology of the samples was investigated using scanning electron microscopy. The Al_2_O_3_ samples developed porous microstructure in the course of the thermal decomposition of the substrate pore former (PU foam) infiltrated with Al_2_O_3_ suspension (Figure 4a). The porous Al_2_O_3_ ceramic forms a continuous network that is homogeneous throughout the sample. The type of porosity can be described as a network where connection points are linked by concave sticks and spherical arch segments. The solid network has sporadic defects, where it can be observed that the solid network seems to be mostly porous with a significantly less porous surface layer (wall thickness of 50 μm) (Figure 4b,c).

**Figure 5:**
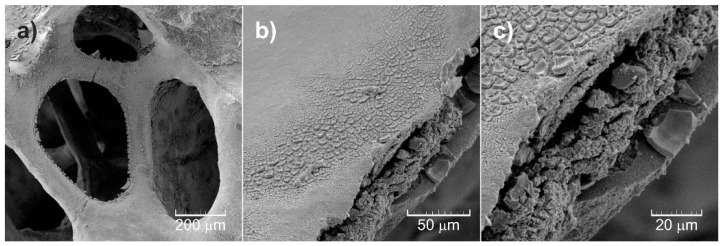
SEM of TiO_2_-coated Al_2_O_3_ foam: (**a**) network, (**b**) surface at high resolution (HR), (**c**) crack pattern of TiO_2_ coating layer visible on micrographs at high magnification.

**Figure 6:**
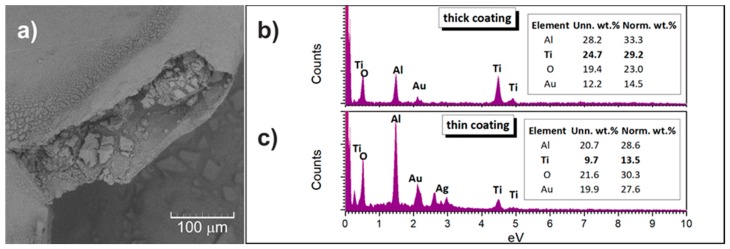
(**a**) SEM using back scatter (BS) electrons on TiO_2_-coated Al_2_O_3_ foam, (**b**) EDS recorded from a wide area of the TiO_2_-coated Al_2_O_3_ foam, (**c**) EDS recorded from a highly magnified area of the TiO_2_-coated Al_2_O_3_ foam with a characteristic crack pattern.

For TiO_2_-coated Al_2_O_3_ foam the morphology configuration remains stable (Figure 5a). The changes in the microstructural parameters at the micro-level are absent. The solid network seems to be coated in uniform manner. The surface was also monitored at higher magnification (Figure 5b). Most of the sites present a uniform TiO_2_ coating and cannot be distinguished from the Al_2_O_3_ network using backscattered electrons (Figure 6a). The composition of the samples was determined using EDS analysis and confirmed to comply with Al, Ti, O from the sample and Ag, Au and C from the tape, glue and conducting layer (Figure 6b,c). Possible presence of carbon residuals as a consequence of the PU burning, as suggested by the DTA/TGA results, cannot be evaluated due to the presence of C from the adhesive tape. Sporadically a cracked pattern on the TiO_2_ surface is observed because of the different drying conditions for different TiO_2_ coating thickness in bulk and surface regions of the TiO_2_-coated Al_2_O_3_ foam porous sample (Figure 5c). The EDS of this area, obviously with thicker TiO_2_ coating, indeed shows stronger presence of Ti (Figure 6b). The SEM analysis allowed an estimation of the TiO_2_ coating thicknesses in the range from 150–300 nm.

### 3.4. Structural Properties of the Al_2_O_3_ Foam Substrate and TiO_2_-coated Al_2_O_3_ Foam

**Figure 7:**
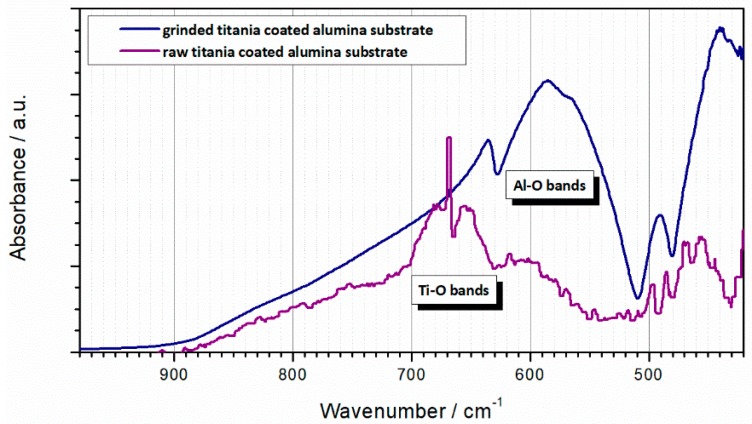
Attenuated total reflectance (ATR) FTIR scans of TiO_2_-coated Al_2_O_3_ foam: raw porous substrate sample (purple line) and ground powdered sample (blue line).

The as-received bulk cube of TiO_2_-coated Al_2_O_3_ foam was scanned in the reflectance mode using FTIR (Figure 7). The main band is observed in the scan between 640 and 700 cm^−1^, as well as slightly below 500 cm^−1^, all assigned to Ti–O stretching vibrations [40]. According to the literature, the large band between 900 and 500 cm^−1^ is characteristic for Al_2_O_3_, where stretching vibration of the Al–O–Al bond occurs. The broadening occurs due to the distribution of vacancies between octahedral and tetrahedral sites and spread thereof of the Al–O vibrational frequencies [41]. Generally, the scan has poor resolution (noisy signal, strong background) due to limited ATR prism coverage because of the high porosity of the sample. No other phases are present except a trace quantity of adsorbed moisture.

In addition, the bulk TiO_2_-coated Al_2_O_3_ foam was ground into powder and subjected to FTIR scan again. The powdered scan yields a better ATR prism coverage, with a clear signal and low background. Qualitatively the presence of the previously assigned bands remains the same; however, the quantitative ratios are different (Figure 7). The TiO_2_ band is barely observed in the powdered sample, reflecting the overall low content of TiO_2_ in the powdered sample. On the contrary, stronger TiO_2_ signal is visible in the as-received bulk cube of TiO_2_-coated Al_2_O_3_ foam sample (stronger than Al_2_O_3_). Namely, using ATR reflectance mode, the signal is predominately collected from the sample surface, allowing relative overestimation of quantity of the coating species. Given that the TiO_2_ is present as a coating on the Al_2_O_3_ foam substrate, the greater TiO_2_ signal indeed a relatively overestimate. Such a relatively strong presence of TiO_2_ in turn confirms the TiO_2_ is indeed forming a coating layer on the surface of the Al_2_O_3_ substrate.

**Figure 8:**
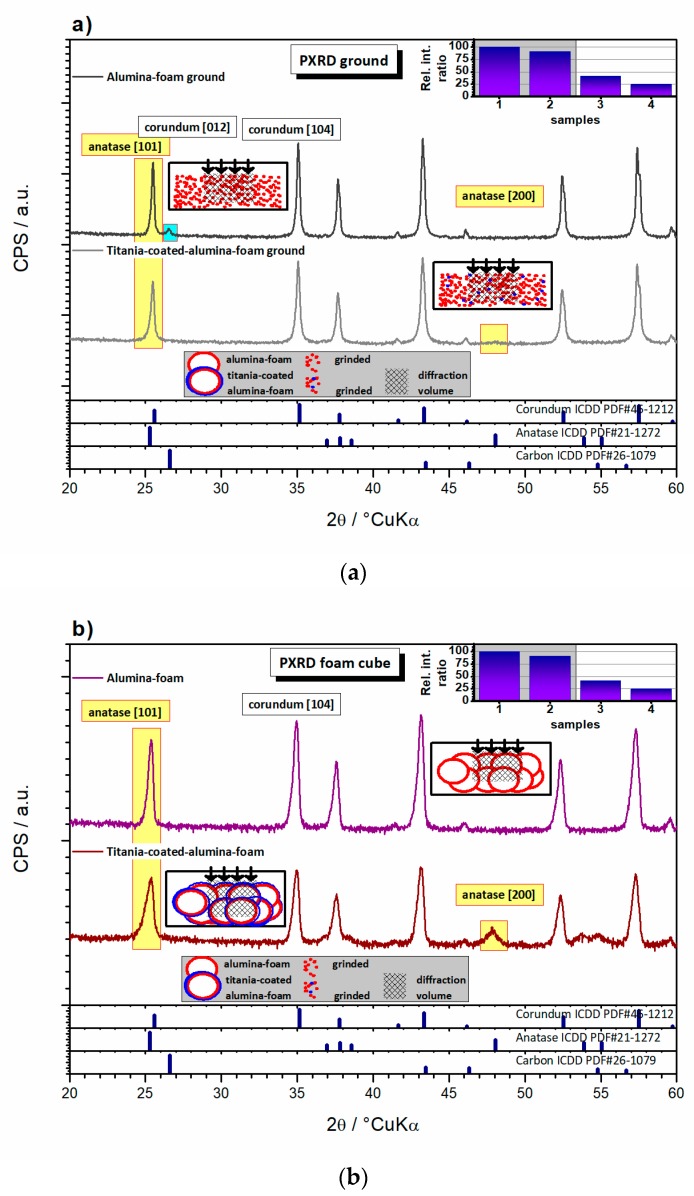

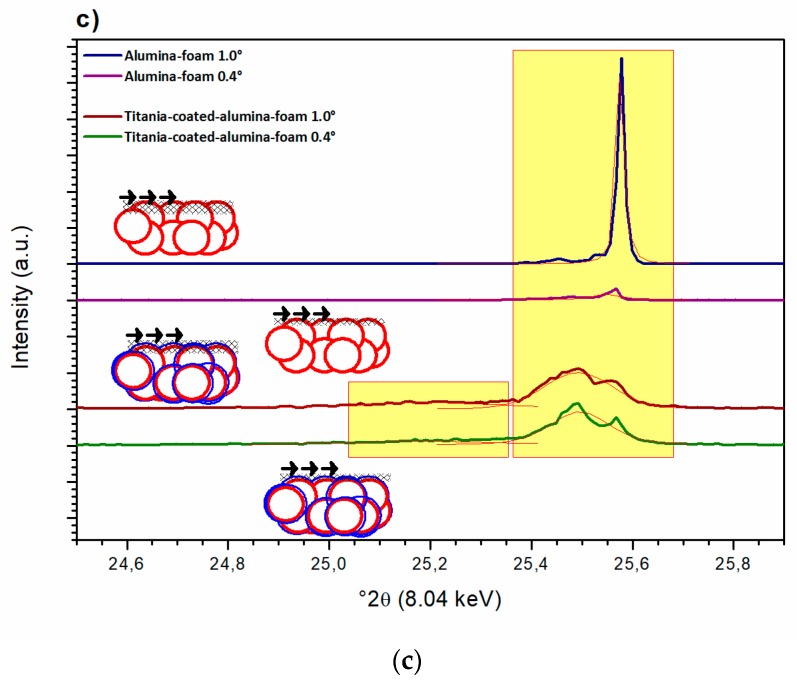
Al_2_O_3_ foam and TiO_2_-coated Al_2_O_3_ foam samples analyzed: (**a**) using Powder X-ray Diffraction (PXRD) on ground powders, (**b**) on bulk cubic samples and (**c**) using synchrotron radiation Grazing Incidence X-ray Diffraction (GIXRD) in thin-film measurement configuration.

A better insight into the structural development of the Al_2_O_3_ phase in the Al_2_O_3_ foam is offered by the XRD analysis (Figure 8a,b). The Al_2_O_3_ foam substrate and the TiO_2_-coated Al_2_O_3_ foam bulk cube samples were ground into powder and XRD analyzed. For both the Al_2_O_3_ foam and the TiO_2_-coated Al_2_O_3_ foam samples, the Al_2_O_3_ phase, assigned to corundum (ICDD PDF#46-1212), was found as the main crystalline phase. In addition to the corundum, the traces of a phase that was assigned to carbon (ICDD PDF#26-1079) were found in the Al_2_O_3_ foam ground sample (Figure 8a). The graphite-like carbon probably occurs as a consequence of the exfoliation of carbon residuals generated during the thermal decomposition of the PU foam template, which is in concordance with the DTA/TGA results. This is the only sample where carbon was observed. The TiO_2_-coated Al_2_O_3_ foam sample was thermally treated twice, which allowed an opportunity for additional thermal decomposition and the removal of the carbon residuals (Figure 8b). The anatase [101] strongline (Irel = 100%) peak at 25.28° 2θ heavily overlaps with the corundum [012] peak (Irel = 45%) at 25.58° 2θ, so anatase presence cannot be confirmed (Figure 8a,b). The anatase [200] peak (I_rel_ = 35%) at 48.05° 2θ does not overlap, but the peak phase assignation on a single weak peak cannot be used to confirm TiO_2_ phases beyond any doubt. It should be pointed out that any thin-film coating, such as TiO_2_-coated Al_2_O_3_ foam, represents a minute volume fraction in powdered samples. Also, XRD is a statistical analysis technique where the given quantitative signal corresponds well to the phases mass fraction (assuming similar absorption coefficients, which is roughly the case for the mentioned oxide systems) in the diffraction volume of the samples. Since the diffraction volume for the used materials (attenuation distance using copper X-ray radiation is approximately 0.3 mm [42]) is huge in comparison to the unit cell dimension, the PXRD analysis on ground samples is definitively not a surface sensitive technique, as shown in schemes in Figure 8a. Considering the abovementioned facts, the absence of a TiO_2_ signal does not come as a surprise, as the TiO_2_ signal is under the detection threshold of the technique.

To increase the surface sensitivity samples in bulk cubes using a special holder can be analyzed. In this case, the diffraction volume is comparable, but the PXRD method is not fully statistical, as shown by schemes in Figure 8a,b. The TiO_2_ phase is not statistically represented through the analyzed sample volume as in the first case, i.e., there is no homogeneous volume distribution, and the surface signal is slightly overestimated, though the method is still not surface sensitive. Due to the fact that TiO_2_ is located on the surface, a vague TiO_2_ signal was detected and it was attributed to the anatase (ICDD PDF#21-1272). The anatase [101] strongline peak at 25.28° 2θ tilts the corundum [012] peak visibly. The [200] peak (Irel = 35%) at 47.83° 2θ was strong enough to allow the Voight function fit to yield Scherrer crystallite size of 36 nm (assuming spherical shape of crystallites), pointing to the nanosized TiO_2_ coating (Figure 8b). In the Al_2_O_3_ foam sample, the carbon phase may be present but is under the detection threshold of PXRD in this configuration. Indirectly, the semi-quantitative presence of the corundum phase is a function of the apparent presence of TiO_2_ coating on the sample surface. Ground TiO_2_-coated Al_2_O_3_ foam thereof shows less corundum than ground Al_2_O_3_ foam, while bulk cube samples showed less corundum due to an increased surface sensitivity (again TiO_2_-coated Al_2_O_3_ foam shows less corundum than Al_2_O_3_ foam) (Figure 8a,b inset).

Both methods are not completely geometrically appropriate for the determination of crystalline thin films. For this purpose, Grazing Incidence X-ray Diffraction (GIXRD) technique is suitable. The measurement is configured to maximize the signal from the surfaces of small samples and thin films as shown by scheme in Figure 8c.

However, this normally refers to the case of planar thin films. In the studied, the TiO_2_ thin film is not planar. Nevertheless, in comparison with the abovementioned two PXRD methods, the grazing configuration additionally enhances the surface signal in the overall signal. The attenuation distance is the same as in the previous case, but at low incidence angles due to the configuration, the attenuation occurs in the surface region, which gives rise to a much smaller diffraction volume (Figure 8b scheme).

Therefore, this method is surface sensitive, and by changing the incidence angles, depth profiling of the crystalline phases is allowed. In the case of the synchrotron GIXRD analysis the beam is very monochromatic, and therefore the peaks are narrow. As a result of that, the signal from anatase [101] and corundum [012] peaks was distinguished, deconvoluted and used to confirm the presence of the anatase coating on corundum foam in the bulk cube sample. Depth profiling at different grazing angles influenced the surface sensitivity and affected the apparent corundum phase quantity. A lower grazing angle favored surface sensitivity and relatively decreased corundum signal, but under more noise. Splitting of the corundum peak occurs as the diffracted signal may be collected from positions in the sample that are out of plane, despite the spot analysis configuration. Quite small difference of the TiO_2_-coated Al_2_O_3_ foam GIXRD signal at 0.4 and 1.0° 2θ points to a thickness of TiO_2_ not less than 100 nm, which is in concordance with the SEM results.

### 3.5. Photocatalytic Degradation of Memantine on TiO_2_-coated Al_2_O_3_ Foam and TiO_2_ Nanoparticles P25

**Figure 9:**
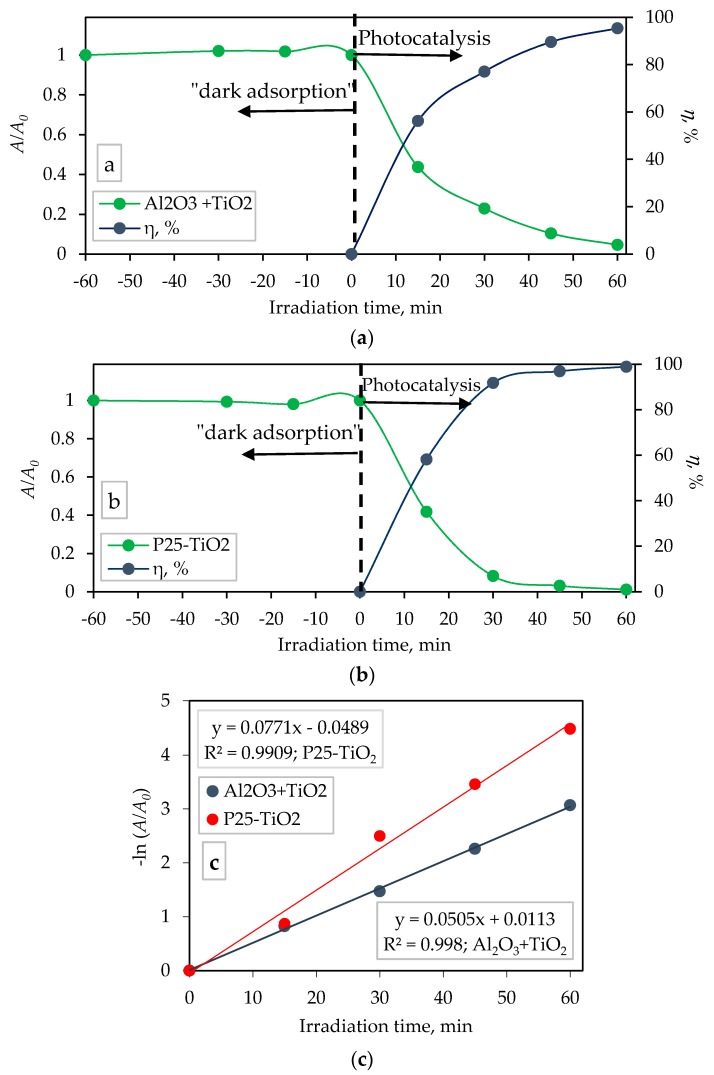
Adsorption and photocatalytic degradation of memantine as a function of irradiation time by: (**a**) TiO_2_-coated Al_2_O_3_ foam, (**b**) suspended TiO_2_ nanoparticles P25 and (**c**) first-order plot of memantine photocatalytic degradation. Time interval marked as “−60 min” to time “0” indicates that the solution was not irradiated in the 60 min period prior to the time the UV-A LED lamp was switched on, i.e., the beginning of photocatalytic experiment. All of the experiments were triplicated with errors below 4%.

The photocatalytic activity of the sol-gel derived TiO_2_ film immobilized on Al_2_O_3_ foam was compared with the commercially available TiO_2_ nanoparticles P25-Degussa in the form of a suspension (100 mg TiO_2_/100 mL memantine solution) under UV-A LED lamp irradiation with a radiation peak at 365 nm. It is important to bear in mind that functional comparison of titania-coated alumina foam would be possible only using an established titania-based immobilized-type of catalyst. However the only recognized titania-based catalyst material is P25 which is a suspension-based catalyst. Therefore we stress that the significance of the comparison is more theoretical and less practical. The degradation profile of the aqueous solution of memantine, as well as the percentage of photocatalytic activity of the TiO_2_ film immobilized on the Al_2_O_3_ foam and in suspension form under a UV-A LED lamp irradiation are shown in Figure 9a,b, respectively.

The percentage of photocatalytic activity of TiO_2_-coated Al_2_O_3_ foam and TiO_2_ nanoparticles P25 was calculated using the equation:

(2) $$\eta ,\;\%=\frac{A_{0}-A_{t}}{A_{0}}\times 100$$

where *η* is the percentage of degradation, *A*_0_ is the chromatographic peak area of the initial memantine concentration before irradiation under UV light and *A_t_* is the concentration of memantine expressed as integrated area of chromatographic peak at sampling time *t* (min).

The memantine degradation followed the first-order kinetics (Figure 9c), represented with the following equation [31,43,44]:

(3) $$A_{t}=A_{0}\cdot e^{-kt}$$

where, *k* (min^−1^) is the degradation rate constant. The first-order degradation rate constant (*k*) from Equation (3) can be calculated by the slope of the straight line obtained from plotting linear regression of −ln(*A*/*A*_0_) versus irradiation time (*t*) (Figure 9c). The correlation coefficient (*R*^2^) was 0.9980 (for experiments with TiO_2_-coated Al_2_O_3_ foam) and 0.9909 (for TiO_2_ nanoparticles P25) which indicates that the degradation of memantine follows the first-order kinetics and this is well represented by the first-order kinetics model.

The half-life (*t*_1/2_) was calculated by following equation [43]:

(4) $$t_{1/2}=\frac{\ln2}{k}$$

The kinetic data obtained in Figure 9c regarding to the pseudo first-order rate constant, *k*, and the half-life, *t*_1/2_, for the photocatalytic degradation of memantine are listed in Table 2.

The percentage of photocatalytic degradation of memantine by TiO_2_-coated Al_2_O_3_ foam after 60 min was 96% (*k* = 0.0505 min^−1^, *t*_1/2_ = 13.73 min; Figure 9a,c and Table 2), while the degradation by commercially available TiO_2_ nanoparticles P25 was 99% (*k* = 0.0771 min^−1^, *t*_1/2_ = 8.99 min; Figure 9b,c and Table 2). During the irradiation, for both conditions, a decrease in the concentration of memantine was observed. The TiO_2_ P25 nanoparticles suspended in the memantine solution showed a higher activity in comparison to the sol-gel derived TiO_2_ film immobilized on the Al_2_O_3_ foam. As expected, the suspension is much more active than the immobilized photocatalyst [45,46]. The higher photoactivity of the suspended TiO_2_ P25 sample can be explained in terms of the availability of active sites on the catalyst surface and the penetration of the UV light into the suspension as a result of an increased screening effect and scattering of light. The radiation wavelength of 365 nm possesses enough energy to activate the photocatalytic oxidation/reduction process on the TiO_2_ surface [47] which results in the degradation of memantine.

In most studies, TiO_2_ as photocatalyst is used in the form of a suspension, which allows an efficient reaction, but the main disadvantage is the difficult removal of TiO_2_ from the reaction mixture after the process is over. The catalyst is usually removed by filtration, which adds an extra step to the whole process. Therefore, the applicability of TiO_2_ in the form of a suspension is not optimal for the applications in real systems. These disadvantages of the application of TiO_2_ nanoparticles in the suspension form may be eliminated by the immobilization of TiO_2_ on different substrates in the form of a stable nanostructured film [29,30,31,48], which was applied in this work. The most widely used method for producing the TiO_2_ film is the sol-gel synthesis process [24,49].

Besides the performed photocatalytic activity experiments, the adsorption of memantine on the surface (“dark adsorption”) of alumina foams (with and without TiO_2_ film) as well as on TiO_2_ P25 nanoparticle was monitored. It was found that the adsorption of memantine was negligible on all investigated surface samples: the surface of TiO_2_ film immobilized on Al_2_O_3_ foam (Figure 9a), suspended TiO_2_ P25 (Figure 9b) as well as on pure alumina foam (alumina foam without TiO_2_, not shown here). Also, the photolytic degradation of memantine under UV-A radiation (365 nm) (without TiO_2_) was negligible (hence was not graphically presented in the paper).

## 4. Conclusions

The PU template was repeatedly immersed in an aqueous Al_2_O_3_ suspension containing 60 wt.% ceramic powder, 0.4 wt.% dispersant Dolapix CE 64, 3.5 wt.% PVA binder and 0.5 wt.% defoaming agent Foamaster MO 2111. After the heat treatment, including burnout of organic matter (between 300 °C and 600 °C) and sintering at 1600 °C, a ceramic replica of the PU template was obtained and used as a substrate for the sol-gel TiO_2_ film deposition.

A combination of structural characterization methods was proposed to qualify and quantify the existence of a crystalline coating on a 3D substrate with uneven surface. Nano-sized TiO_2_ film was identified as anatase phase on corundum foam substrate, using advanced diffraction setups on bulk and ground samples.

Sol-gel derived TiO_2_ film immobilized on Al_2_O_3_ foam was used for the photocatalytic degradation of aqueous memantine solution under LED lamp irradiation with a radiation peak at 365 nm. The results demonstrated that the photocatalytic memantine degradation followed the first-order kinetics, with degradation rate constant and half-life of 0.0505 min^−1^ and 13.73 min, respectively. Memantine photocatalytic degradation of 96% was achieved after 60 min of LED lamp irradiation.

The photocatalytic activity of the sol-gel derived TiO_2_ film immobilized on Al_2_O_3_ foam was compared with commercially available TiO_2_ nanoparticle P25-Degussa in the form of a suspension. The results demonstrated that the photocatalytic memantine degradation by suspended TiO_2_ P25 nanoparticles also followed the first-order kinetics, with a higher degradation rate constant of 0.0771 min^−1^ and lower half-life of 8.99 min. The percentage of photocatalytic degradation of memantine by commercially available TiO_2_ P25 nanoparticles after 60 min was higher (99%) than for the experiment with the immobilized film.

From a practical point of view the immobilized TiO_2_ is much more suitable for use as a catalyst because there is no need for the separation of nanoparticle after the photocatalytic degradation process.

It can be concluded that the combined replica and sol-gel methods were successfully used to prepare a valuable porous photocatalyst with suitable structural and mechanical properties for application as a memantine-degrading catalyst.

## Figures and Tables

**Table 1:** Properties of Al_2_O_3_ foam substrate and TiO_2_-coated Al_2_O_3_ foam.

	*ρ*, g/cm^3^	*ϕ*, %	Strut Thickness, µm	Pore Size, µm	*σ*, MPa
Al_2_O_3_	0.46 ± 0.03	88.50 ± 0.76	52.73 ± 4.79	430.14 ± 52.49	2.03 ± 0.17
TiO_2_-Al_2_O_3_	0.44 ± 0.02	88.96 ± 0.70	57.46 ± 5.14	436.01 ± 57.02	2.04 ± 0.19

**Table 2:** The first-order degradation rate constant (*k*) and half-life (t_1/2_) for memantine photodegradation by TiO_2_-coated Al_2_O_3_ foam and suspended TiO_2_ nanoparticles P25-Degussa.

Photocatalyst	*k*, min^−1^	*t*_1/2_, min	*R* ^2^
TiO_2_-coated Al_2_O_3_ foam	0.0505	13.73	0.9980
TiO_2_ P25-Degussa	0.0771	8.99	0.9909
